# Eicosanoids in Cancer: Prostaglandin E_2_ Receptor 4 in Cancer Therapeutics and Immunotherapy

**DOI:** 10.3389/fphar.2020.00819

**Published:** 2020-05-29

**Authors:** Mc Millan Ching, Jocelyn Reader, Amy M. Fulton

**Affiliations:** ^1^Cellular and Molecular Medicine, Johns Hopkins University School of Medicine, Baltimore, MD, United States; ^2^Division of Gynecologic Oncology, Department of Obstetrics, Gynecology and Reproductive Sciences, University of Maryland School of Medicine, Baltimore, MD, United States; ^3^University of Maryland Marlene and Stewart Greenebaum Comprehensive Cancer Center, University of Maryland, Baltimore, Baltimore, MD, United States; ^4^Department of Pathology, University of Maryland School of Medicine, Baltimore, MD, United States; ^5^Baltimore Veterans Administration Medical Center, Baltimore, MD, United States

**Keywords:** EP4, microenvironment, prostaglandin E_2_, immunotherapy, cancer

## Abstract

The cyclooxygenase-2 (COX-2) enzyme is frequently overexpressed in epithelial malignancies including those of the breast, prostate, lung, kidney, ovary, and liver and elevated expression is associated with worse outcomes. COX-2 catalyzes the metabolism of arachidonic acid to prostaglandins. The COX-2 product prostaglandin E_2_ (PGE_2_) binds to four G-protein-coupled EP receptors designated EP1–EP4. EP4 is commonly upregulated in cancer and supports cell proliferation, migration, invasion, and metastasis through activation of multiple signaling pathways including ERK, cAMP/PKA, PI3K/AKT, and NF-κB. EP4 antagonists inhibit metastasis in preclinical models. Cancer stem cells, that underlie therapy resistance and disease relapse, are driven by the expression of EP4. Resistance to several chemotherapies is reversed in the presence of EP4 antagonists. In addition to tumor cell-autonomous roles of EP4, many EP4-positive host cells play a role in tumor behavior. Endothelial cell-EP4 supports tumor angiogenesis and lymphangiogenesis. Natural Killer (NK) cells are critical to the mechanism by which systemically administered EP4 antagonists inhibit metastasis. PGE_2_ acts on EP4 expressed on the NK cell to inhibit tumor target cell killing, cytokine production, and chemotactic activity. Myeloid-derived suppressor cells (MDSCs), that inhibit the development of cytotoxic T cells, are induced by PGE_2_ acting on myeloid-expressed EP2 and EP4 receptors. Inhibition of MDSC-EP4 leads to maturation of effector T cells and suppresses the induction of T regulatory cells. A number of EP4 antagonists have proven useful in dissecting these mechanisms. There is growing evidence that EP4 antagonism, particularly in combination with either chemotherapy, endocrine therapy, or immune-based therapies, should be investigated further as a promising novel approach to cancer therapy. Several EP4 antagonists have now progressed to early phase clinical trials and we eagerly await the results of those studies.

## EP4 in the Malignant Cell

The cyclooxygenase-2 (COX-2) enzyme catalyzes the rate-limiting step in the metabolism of arachidonic acid to prostaglandins. COX-2 is frequently overexpressed in epithelial malignancies and is associated with poor clinical outcomes. In cancer, the most abundant COX-2 product is prostaglandin E_2_ (PGE_2_) which binds four G-protein-coupled EP receptors (EP1–EP4) to activate several intracellular signaling pathways. EP4 is upregulated and contributes to the pathology of many human malignancies including those of the breast, prostate, colon, ovary, and lung (Reader et al., 2011; Majumder et al., 2018; Take et al., 2020). EP4 receptor signaling is linked to many properties of malignant cells including proliferation, migration, invasion, metastasis, angiogenesis, immune evasion, epithelial to mesenchymal transition, and cancer stem cell (CSC) properties. This review will summarize some of the growing evidence that EP4 contributes to cancer progression both in a tumor cell-autonomous manner and by modulating the behavior of EP4-positive host cells in the tumor microenvironment.

### The Role of EP4 in Cell Proliferation

Heightened EP4 expression supports the proliferation of some, but not all malignancies. Many mechanisms contribute to the growth-promoting properties of the PGE_2_/EP4 axis. Exposure of cultured cancer cell lines to PGE_2_ or specific EP4 agonists can lead to modest stimulation of cell growth; conversely, small molecule EP4 antagonists or EP4 gene targeting inhibits proliferation of some cell lines. For example, overexpression of EP4 in prostate cancer cells results in increased proliferation *in vitro* and in xenograft models (Terada et al., 2010). The pro-proliferative response is associated with the cAMP/PKA/PI3K-Akt signaling pathway (Xu et al., 2018). These findings support the continued investigation of EP4 as a potential target in castration-resistant prostate cancer. Mice transgenic for epithelial EP4 overexpression display more squamous cell carcinomas (Simper et al., 2014). An exception to the general conclusions regarding the tumor-promoting role of EP4 is in gastric carcinoma cell lines where administration of EP2 and EP4 *agonists* resulted in growth inhibition, decreased cell proliferation, and was accompanied by cAMP production. The possible role of EP antagonists was not investigated (Okuyama et al., 2002).

Elevated EP4 expression drives COX-2 expression and PGE_2_ secretion in uterine cervical cancer tissue, promoting colony formation and VEGF expression (Oh et al., 2009). In colorectal cancer, EP4 occupation leads to ERK activation supporting anchorage-independent growth and resistance to apoptosis that is reversed by small molecule EP4 antagonists ONO-AE3-208 and AH23848 (Hawcroft et al., 2007). Likewise, inhibition of the EP2 and EP4 receptors (with AH6809 and GW627368X, respectively) represses IGF-1-induced proliferation of pancreatic BxPC-3 cancer cells (Takahashi et al., 2019) and is accompanied by increased phospho-PKC-θ and decreased phospho-ERK (Takahashi et al., 2015).

### The Role of EP4 in Cell Migration/Invasion/Metastasis

Tumor dissemination is the chief cause of cancer mortality. Several early studies demonstrated that small molecule EP4 antagonists (AH23848; ONO-AE3-208) or EP4 gene silencing reduced metastatic potential *in vivo* in preclinical models of breast, prostate, colon, and lung cancer (Ma et al., 2006; Yang et al., 2006; Xu et al., 2018). The anti-metastatic activity is partially attributed to direct inhibition of tumor cell migration and invasion. For example, EP4-shRNA knockdown in lung cancer cells led to decreased cell migration *in vitro* by a β-arrestin1-dependent mechanism (Kim et al., 2010). EP4 blockade in prostate cancer cells overexpressing EP4 resulted in reduced migration, invasion, and metastasis. Down-regulation of EP4 and EP2 receptors or the EP4 antagonist AH23848 inhibit migration and invasion of human colorectal carcinoma cells (Jeong et al., 2018). Conversely, agonism of EP4 promoted *in vitro* lung cancer cell migration (Kim et al., 2010). EP4 is coupled to several downstream signaling pathways. In prostate, colon, and renal cell carcinomas, EP4 supports cell proliferation and invasion *via* the cAMP-PKA/PI3K-AKT signaling pathway and this response is inhibited by L161982 (Zhang et al., 2017) by ONO-AE3-208 or Cayman 10598 (Kashiwagi et al., 2018) or by RQ15986 (Majumder et al., 2018). EP4 regulates cell migration through Orai1 Ca^2+^ signaling in human oral squamous carcinoma cell lines that is blocked by ONO-AE3-208; cancer metastasis was inhibited when EP4 gene expression was reduced (Osawa et al., 2020). In melanoma, EP4 agonism induces cell migration accompanied by accumulation of β-catenin and decreased expression of several metalloproteinases (Vaid et al., 2015). Knockdown of EP4 abolished the transendothelial migration and metastatic intravasation capacity in metastatic renal carcinoma (Zhang et al., 2017). EP4 agonists can induce and the EP4 antagonist GW627368x blocks EGFR-dependent degradation of the extracellular matrix that, if left unchecked, facilitates breast cancer invasion (Tönisen et al., 2017).

The COX2/EP4 pathway is coupled to induction of several proinflammatory cytokines; several are tumor-promoting. In prostate cancer, the inhibitory effects of the EP4 antagonist AH23848 were linked to downregulation of several cytokines (CCL2, IL6, and CXCL8) (Han et al., 2019). In a murine model of bone metastatic prostate cancer, the EP4 antagonist ONO-AE3-208 abrogated inflammation-dependent bone metastasis and bone loss (Watanabe et al., 2016). EP4 antagonism with the same compound also prevents bone loss in malignant melanoma cells *via* the suppression of osteoclasts (Takita et al., 2007).

### Cancer Stem Cells and EP4 in Chemoresistance

More recently, EP4 has been implicated in treatment resistance and as a driver of CSCs. Epigenetic activation of the EP4 receptor may support resistance to endocrine therapy in patients with breast cancer that is reversed by GW627368x (Hiken et al., 2017). Several labs have shown that EP4 is upregulated in malignant cells with stem-like properties and that treatment of tumor-bearing mice with EP4 antagonists (RQ15986, AH23848, Frondoside A) reduces the number of CSC (Kundu et al., 2014; Majumder et al., 2014). A positive feedback loop involving EP4/miR-526b and miR-655/COX-2 is critical to the mechanism by which EP4 supports breast CSC (Majumder et al., 2018). Exposure of MCF7 cells to EP4 agonists upregulated both miR species and was accompanied by activation of PI3K/AKT, ERK, and NF-κB pathways leading to increased cell proliferation, migration, invasion, spheroid formation, and epithelial to mesenchymal transition. EP4 can mediate the effects of hypoxia-driven mesenchymal stem cells in the progression of hepatocellular carcinoma. This response is reversed in EP4 siRNA expressing cells or in the presence of GW627368x (Liu et al., 2019). The EP4 antagonist L161982 blocks the ability of enteric glial cells to stimulate colon CSCs *via* a PGE2/EP4/EGFR-dependent pathway (Valès et al., 2019). Although the exact mechanism by which EP4 antagonism allows for various cancer cells to transition from the mesenchymal/CSC state to an epithelial phenotype is not clear, one group has proposed that EP4 antagonism with GW627368x results in extracellular vesicle-mediated clearance of CSC markers, integrins, and drug transporters (Lin et al., 2018).

There is growing interest in the potential of EP4 antagonists in combination with other therapies, including chemotherapy. Gynecologic uterine smooth muscle tumors are sensitized to docetaxel in the presence of the EP4 antagonists AH23848 or RQ15986 (Reader et al., 2019). In a model of oxaliplatin-resistant colon cancer, EP4 blockade with L161982 inhibited CSC markers and tumorsphere formation (Huang et al., 2019). Likewise, cisplatin-resistant gastric cancer cells are sensitized to cisplatin *via* the suppression of COX-2/EP4/ERK1/2/P38 signaling by L161982 (Lin et al., 2019).

## The Role of EP4 in the Host Response to Malignancy

### Regulation of Lymphangiogenesis

In addition to tumor cell-autonomous roles for EP4, many host cells express EP4 and should be considered in the context of systemic EP4 targeting. EP4 plays a role in activation of PGE_2_ and COX-2 expression in rat mesenteric lymphatic endothelial cells (Nandi et al., 2017). EP4 antagonism with RQ15986 inhibits tumor growth and angiogenesis in a murine model of breast cancer (Majumder et al., 2014). In melanoma, PGE_2_ acts on EP4 expressed on fibroblasts in the tumor microenvironment to regulate the release of angiogenic factors (Inada et al., 2015).

### Regulatory Function of EP4 on NK Cells

Innate immunity plays a significant role in tumor control. Natural killer cells recognize and directly kill malignant cells and provide critical cytokines that promote the replication and activation of cytotoxic T lymphocytes. There are also critical interactions between NK and dendritic cells that further drive both innate and adaptive anti-tumor responses. Each of these functions is regulated by PGE_2_ acting on EP receptors expressed on the NK cell. While NK cells express each EP, the negative regulation by PGE_2_, in the context of malignancy, is through EP4 and EP2 (Kundu et al., 2009; Holt et al., 2012; Ma et al., 2013). EP4 antagonists AH23848, ONO-AE3-208, Frondoside A and RQ15986, or to a lesser extent, the EP2 antagonist AH6809, prevent PGE_2_-mediated inhibition of NK cells.

In preclinical models of triple negative breast cancer, even when EP4 is directly inhibited in the malignant cell by shRNA targeting, the anti-metastatic activity is lost when NK cells are depleted in the host (Kundu et al., 2009; Ma et al., 2013). NK cells isolated from either patients or tumor-bearing mice are compromised regarding the ability to lysis target cells, produce critical cytokines and to migrate in response to chemotactic signals. NK recognition and lytic activity is triggered by the presence of activating ligands and the loss of inhibitory signals on the target cells; the latter are delivered chiefly through the expression of MHC class I antigens on the target cell. EP4 antagonists including Frondoside A, reduce surface expression of MHC class I antigens and, at the same time, increase sensitivity to NK-mediated lysis (Holt et al., 2012). PGE_2_ prevents NKG2D, NCR, and CD16 activating receptors from triggering lysis (Martinet et al., 2010). Thyroid cancer-derived PGE_2_ inhibits NK maturation as well as the expression of the NK receptors NK44, NK30, TRAIL, and NKG2D whereas NK inhibitory receptors are increased (Park et al., 2018). Either EP2 (AH6809) or EP4 (AH23848) antagonists block the inhibitory actions of PGE_2_ on NK cells. Administration of EP4 antagonists (RQ15986, AH23848, Frondoside A) to tumor bearing mice restores the profound suppression of NK activity (cytotoxic functions, cytokine production, chemotactic activity), even in the context of progressive tumor growth (Holt et al., 2012; Ma et al., 2013). Thus, in both murine and human NK cells, the activating signals for lysis are reduced and the inhibitory signals are enhanced in response to PGE_2_ and these inhibitory signals are reversed by EP4 blockade. Importantly, the ability of NK cells to control metastasis is restored.

In addition to direct inhibitory effects of tumor-PGE_2_ on NK activities, myeloid-derived suppressor cells (MDSC) can also negatively regulate NK functions. MDSC derived from patients with advanced melanoma inhibit the activity of co-cultured NK cells (Mao et al., 2014). PGE_2_, acting on EP2 and EP4 receptors on the MDSC, activates p38 MAPK/ERK pathways leading to secretion of TGFβ to inhibit NK cells. This mechanism of immune suppression is blocked by AH6809 or AH23848. The critical role of PGE_2_ in regulating interactions between dendritic cells and NK cells was further elucidated in a recent study (Böttcher et al., 2018). In addition to MDSC regulating NK cells, NK cells can, in turn, recruit conventional type 1 dendritic cells that are critical to immune-mediated tumor control. cDC1 are recruited by the release of CCL5 and XCL1 from NK cells. The ability of PGE_2_ to compromise NK functions also results in impaired recruitment of cDC1 to the tumor microenvironment in a COX-2 dependent manner.

### MDSC and M2 Macrophages Are Induced Through PGE2/EP Activation

MDSC can become the dominant immune cell population in progressively growing tumors. PGE_2_ induces bone marrow stem cells to differentiate into Gr1+CD11b+ MDSCs but either EP2 (AH6809) or EP4 (AH23848) antagonists block the induction (Sinha et al., 2007). In several tumor models, an EP4 antagonist (E7046) similarly blocked the induction of MDSC and M2 macrophages *in vivo* (Albu et al., 2017). Novel mechanisms by which PGE_2_/EP4 induces MDSC have been reported (Rodríguez-Ubreva et al., 2017). PGE_2_ upregulates DNA methyl transferase 3A (DNMT3A) resulting in hypermethylation and repression of immune-promoting genes, specifically in the MDSC population. Conversely, inhibition of DNMT3A results in loss of this pattern of hypermethylation and loss of the immune suppressive properties of MDSC. The ability of PGE_2_ to induce DNMT3A and subsequent hypermethylation in MDSC was blocked in the presence of either EP2 (PF 04418948) or EP4 (L161982) inhibitors. Doxorubicin-resistant mammary tumor cells have higher levels of COX-2 and PGE_2_ secretion than the Dox-sensitive parental cells (Rong et al., 2016). These resistant cells more effectively induce MDSC and this induction is blocked by EP4 (ONO-AE3-208) or EP2 (AH6809) inhibitors. The microRNA miR-10a is key to the mechanism by which PGE_2_ induces MDSC through a PKA/AMPK-dependent signaling pathway.

PGE_2_ exerts anti-inflammatory activity on macrophages and monocytes. The EP4 antagonists L161,982 or CJ042794 inhibit LPS-induced production of TNFα by lung macrophages (Gill et al., 2016) or by expression of EP4 siRNA in human monocytic cells (Kashmiry et al., 2018). The acute inflammatory response is supported by M1 macrophages whereas resolution of inflammation is driven by M2 phenotype macrophages. PGE_2_ plays a key role in the polarization of macrophages to the M2 phenotype resulting in poor induction of anti-tumor immunity. MicroRNA-21 negatively regulates M2 polarization by PGE_2_ (Wang et al., 2015).

### Actions of EP Blockade on Cytotoxic T Cells and T Regulatory Cells

Mammary epithelial COX-2-derived mediators contribute to pro-tumor immune function, particularly through T lymphocyte and cytotoxic immune cell function (Markosyan et al., 2013). EP4 inhibitors, as monotherapy, do not typically result in marked inhibition of tumor growth in preclinical models of breast, colon, or pancreatic cancer, among others. The modest inhibition of growth of breast tumors is, however, dependent on CD8+ T cells (Albu et al., 2017). The mechanism is not by direct actions of the EP4 inhibitor E7046 on either CD8+ T cells or on T regulatory cells; rather it is likely explained by blockade of MDSC actions. The demonstration that EP4 blockade appears to synergize with checkpoint inhibitor blockade with anti-CTLA-4 antibody or, alternatively, with direct targeting of T regulatory cells with an IL-2/diphtheria toxin fusion product is very promising. The latter combination therapy resulted in an inflamed tumor microenvironment expressing T cell activating and T cell recruiting chemokines including CXCL9, CXCL10, CXCL11, and Ccl5/RANTES and MIP-1α.

## Future Directions

There is growing evidence that EP4 represents a novel therapeutic target in cancer, both expressed on the malignant cell as well as on EP4-positive host cells. It is likely that EP4 antagonists would be used in combination with either cytotoxic therapies or with other immune-modulating strategies (for excellent reviews see Majumder et al., 2018; Take et al., 2020). There is growing evidence that EP4 blockade can reverse chemotherapy resistance in several tumor types and therefore combinations of EP4 antagonists with standard of care chemotherapy should be considered. Combinations of EP4 inhibitors with immune therapies are also being considered. NK cells are particularly important in the control of metastatic disease and, in preclinical models, EP4 antagonists reduce tumor dissemination by an NK-cell-dependent mechanism. Primary tumor control appears to be more dependent on CD8+ T cells. These studies and others suggest that EP4 inhibitors may be more effective when combined with immune based therapies rather than as monotherapy. In support of future combination clinical trials, Albu et al. showed that EP4 antagonism combined with either Treg depletion or with an antibody to CTLA-4 provided superior tumor control to either monotherapy (Albu et al., 2017). Cancer clinical trials to evaluate EP4 inhibitors are recruiting in trials of Grapiprant (ARY-007) in combination with pembrolizumab in subjects with either advanced MSS colorectal cancers (NCT03658772) or, in a separate trial in advanced NSCLC (NCT03696212) (Mizuno et al., 2019; Take et al., 2020). E7046 is being examined in a rectal cancer trial (NCT03152370) and BMS 986310 plus nivolumab is being investigated in a phase 1 trial in advanced solid tumors (NCT03661632). The possible role of EP4 in mediating resistance to endocrine therapies in breast cancer should also be explored further. Based on the ability of EP4 antagonists to inhibit CSCs and chemotherapy resistant cell lines, it is anticipated that clinical trials in these settings will also be forthcoming.

## Author Contributions

MC and AF drafted the manuscript. MC, AF, and JR contributed to manuscript revision, read and approved the submitted version.

## Funding

Studies in AF’s laboratory was supported by the Department of Health and Human Services (USA); U.S. Department of Defense; U.S. Veterans Administration and the Maryland Department of Health. While at the University of Maryland, Baltimore, MC was supported by the STAR-PREP program of the National Institute of General Medical Sciences.

## Conflict of Interest

The authors declare that the research was conducted in the absence of any commercial or financial relationships that could be construed as a potential conflict of interest.
